# Thoracoscopic plication for a huge thoracic meningocele in a patient with Neurofibromatosis

**DOI:** 10.1186/1749-8090-9-85

**Published:** 2014-05-13

**Authors:** Heng-Chung Chen, Ping-Hsien Chang, Shang-Wun Jhang, Bing-Yen Wang

**Affiliations:** 1 Division of Thoracic Surgery, Department of Surgery, Changhua Christian Hospital, No. 135, Nanxium St, Changhua City, Changhua Country 500, Taiwan; 2 Division of Neurosurgery, Department of Surgery, Changhua Christian Hospital, Changhua, Taiwan; 3 School of Medicine, National Yang-Ming University, Taipei, Taiwan; 4 Institute of Medicine, Chung Shan Medical University, Taichung, Taiwan

**Keywords:** Cyst, Thoracic meningocele, Thoracoscopic plication

## Abstract

Intrathoracic meningoceles associated with neurofibromatosis type I are rare, and the optimal treatment is still unknown. Herein, we present the case of a 48-year-old Asian female with a huge thoracic meningocele associated with cutaneous neurofibromatosis type I and kyphoscoliosis of the thoracic spine. The large thoracic meningocele was successfully treated through thoracoscopic plication.

## Background

The first case of an intrathoracic meningocele was reported by Phol in 1933 [1]. Patients with intrathoracic meningoceles frequently have neurofibromatosis type I. The clinical symptoms of intrathoracic meningoceles vary, and the initial presentation may include cough, dyspnea, headache, or paraparesis [2]. Herein, we report the case of an Asian female with a huge intrathoracic meningocele and neurofibromatosis type I successfully treated by thoracoscopic plication.

## Case presentation

**Figure 1:**
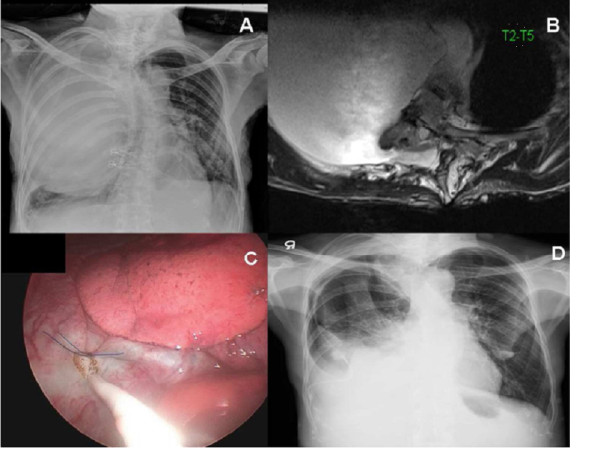
**Chest plain radiograph findings before and after 3 months of thoracoscopic plication. (A)** A large opaque shadow was seen in the right lung field with atelectasis of the lower lobe and kyphoscoliosis of thoracic spine was marked. **(B)** T2-weighted magnetic resonance imaging showed cerebrospinal fluid signal intensity and a meningeal cystic lesion protruding through the right neural foramen at T4. **(C)** The catheter was inserted into the meningocele, and the purse-string suture was tied under thoracoscopic guidance. **(D)** Postoperative chest radiograph at 3 months later showed marked regression of the intrathoracic meningocele.

A 48-year-old Asian female was seen because of radicular pain over the right hemithorax, and progressive dyspnea for 5 days. She was diagnosed with neurofibromatosis type I more than 10 years prior, and also had a moderate thoracic vertebral deformity. The patient was confined to bed or chair for more than 50% of her waking hours. Chest plain radiography showed a right, huge cystic lesion (21 × 11 cm) and marked kyphoscoliosis of the thoracic spine (Figure 1A). Magnetic resonance imaging (MRI) revealed widening of the right 4th, 5th, and 6th neuroforeman (Figure 1B). After discussion with the patient, a decision was made to perform thoracoscopic cystoperitoneal shunt implantation instead of total extirpation through a thoracotomy and laminectomy.

The patient was placed in the left semi-decubitus position and one lung ventilation was begun after induction of general anesthesia. A 30° thoracoscope was placed at the 9th intercostal space in the mid-axillary line. Two 5-mm accessory ports were placed at the 6th intercostal space in the anterior and posterior axillary line, respectively. Under thoracoscopic guidance, a small incision was made at the dependent part of the meningocele, and a purse-string was placed around the incision. A catheter was inserted into the meningocele, and the purse-string suture was tied tightly to avoid migration (Figure 1C). Another 2 cm abdominal incision was created superior to the umbilicus, and a peritoneal catheter was inserted into the peritoneal cavity.

**Figure 2:**
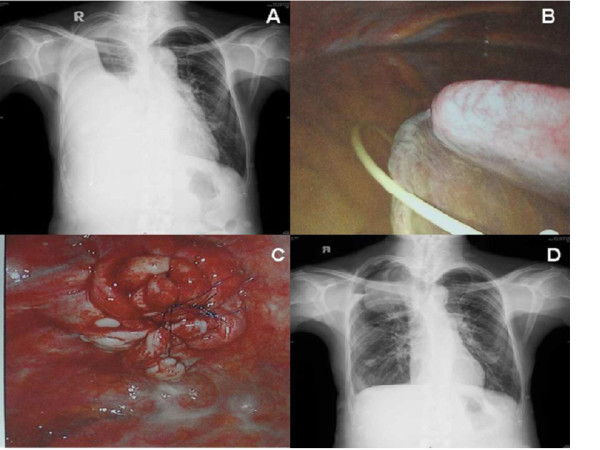
**Chest plain radiograph after thoracoscopic plication. (A)** Right massive and loculated pleural effusions were seen. **(B)** Massive pleural effusion and dislocation of cystoperitoneal shunt were found under thoracoscope examination. **(C)**The meningocele was reduced to a lump of soft tissue at the root with multiple plication. **(D)** Postoperatively chest radiograph at 2 months showed a marked improvement in lung volume.

Her symptoms of dyspnea improved gradually, and at 3 months postoperatively chest radiograph showed marked regression of the intrathoracic meningocele (Figure 1D). At 9 months postoperatively, the patient experienced shortness of breath, and chest plain radiograph revealed a right massive pleural effusion (Figure 2A). A diagnosis of cystoperitoneal shunt dysfunction was considered, and thoracoscopic surgery was arranged.

The patient was positioned in a lateral decubitus position opposite to side of the lesion after induction of general anesthesia and double-lumen endotracheal intubation. A thoracoscope was placed at the 9th intercostal space in the mid-axillary line. A 5-mm accessory port was placed at the 6th intercostal space in the anterior axillary line, and another 5-mm accessory port was placed at the same intercostal space in the posterior axillary line. Thoracoscopic visualization revealed a massive pleural effusion of approximately 2,000 mL and dislocation of cystoperitoneal shunt (Figure 2B). The tip of cystoperitoneal shunt was located in the pleural space, not in the meningocele. The cystoperitoneal shunt was removed, and intrathoracic meningocele plication with 3–0 Prolene suture under thoracoscopic guidance was performed. After plication, the meningocele was reduced to a small mass of soft tissue (Figure 2C). Multiple suture with non-absorbable filaments were placed to maintain the shape of cyst. Her shortness of breath improved significantly after surgery, and chest radiograph at 2 months postoperatively showed a marked improvement in lung volume (Figure 2D).

## Conclusions

The presence of a thoracic meningocele in association with neurofibromatosis type I occurs in approximately 64% of cases [2]. Size can vary, and large meningoceles can occupy the entire hemithorax, as in our case. In some cases, asymptomatic thoracic meningoceles can be treated conservatively with periodic observation. If symptoms are present, surgery should be considered [3]. Various surgical procedures can be performed according to the size of the cyst and include total extirpation through a thoracotomy, laminectomy, or costotransversectomy [3]. These methods are associated with significant risk, and can cause spine instability. Vanhauwaert et al. [4] reported the first cystoperitoneal shunt for an intrathoracic meningocele, and placed the proximal catheter by transthoracic puncture with a Tuohy needle. Tanaka et al. [5] described a similar treatment performed under local anesthesia. In our case, we first performed a thoracoscopic-assisted shunt placement between the meningocele and the peritoneal cavity with placement of a lumboperitoneal valve. Under thoracoscopic guidance, the proximal catheter was placed at the dependent part of intrathoracic meningocele to improve the drainage function. In addition, we also placed a purse-string suture around the entrance of catheter to fix the catheter and avoid dislocation of shunt. Migration of the proximal catheter can result in cerebrospinal fluid leakage and massive pleural effusion. Unfortunately, dislocation of cystoperitoneal shunt still occurred. We then performed thoracoscopic plication of cyst to decrease the volume of cyst, and the lung successfully re-expanded after the procedure. The simple method could be an alternative valuable treatment of pulmonary intrathoracic meningoceles.

Treatment of huge intrathoracic meningoceles in patients with progressive dyspnea can be challenging. Our case demonstrates that thoracoscopic plication is a safe and effective method.

## Consent

Written informed consent was obtained from the patient for publication of this case report and any accompanying images.

## Abbreviations

MRI: Magnetic resonance imaging.

## Competing interests

The authors declare that they have no competing interests.

## Authors’ contributions

HCC and BYW performed the operations and wrote the manuscript. PHC collected the clinical data. SYJ provided the technical support. All authors read and approved the final manuscript.
